# Leveraging Smartphone Mobility Data to Understand HIV Risk Among Rural South African Young Adults: Feasibility Study

**DOI:** 10.2196/67519

**Published:** 2025-08-25

**Authors:** Thulile Mathenjwa, Elphas Luchemo Okango, Khai Hoan Tram, Maxime Inghels, Diego Cuadros, Hae-Young Kim, Fiona Walsh, Till Barnighausen, Adrian Dobra, Frank Tanser

**Affiliations:** 1 Africa Health Research Institute, Krith Building, Third Floor, 719 Umbilo Rd, Durban, 4001, South Africa, 27 315210455; 2 Faculty of Health Sciences, School of Public Health, Division of Epidemiology & Biostatistics, University of Cape Town, Cape Town, South Africa; 3 Department of Medicine, University of Washington, Washington, WA, United States; 4 Lincoln International Institute for Rural Health, University of Lincoln, Lincoln, United Kingdom; 5 Centre Régional de Recherche et de Formation à la Prise en Charge Clinique de Fann, Hôpital Fann, Dakar, Senegal; 6 Digital Epidemiology Laboratory, University of Cincinnati, Cincinnati, OH, United States; 7 Department of Population Health, New York University School of Medicine, New York, NY, United States; 8 Medical Faculty and University Hospital, Heildelberg University, Heidelberg Institute of Global Health, Heidelberg, Germany; 9 Department of Statistics, University of Washington, Washington, WA, United States; 10 Centre for Epidemic Response and Innovation, School of Data Science and Computational Thinking, Stellenbosch University, Stellenbosch, South Africa; 11 South African Centre for Epidemiological Modelling and Analysis (SACEMA), School of Data Science and Computational Thinking, Stellenbosch University, Stellenbosch, South Africa

**Keywords:** geographic mobility, migration, HIV, global positioning system, smartphone, young people, GPS

## Abstract

**Background:**

Smartphones provide a precise method to study human mobility at an unprecedented scale, allowing researchers to explore the links between mobility, HIV risk, and treatment outcomes. However, leveraging smartphone technology to study HIV risk in rural settings presents unique challenges and opportunities.

**Objective:**

This study assessed the feasibility of using smartphone GPS technology to collect mobility data from young adults in rural KwaZulu Natal, South Africa. We also present key lessons learned during the study.

**Methods:**

The study was conducted in 2 phases (June 2021-May 2023) with males and females aged 20‐30 years old. In phase I, participants received smartphones with a customized study app (Avicenna research software). In phase II, they used their personal smartphones and installed the study app. The app used Android location services to record the smartphone location every 30 minutes and send it to a secure study server hourly. Participants were followed up for 6 months (26 wk). If location data were missing for 48‐72 hours, participants were contacted for troubleshooting. Engagement strategies, including reverse billing and gamification (Wheel of Fortune), were implemented to address internet connection barriers and aid data collection.

**Results:**

A total of 207 participants were enrolled (phase I: 163; phase II: 44) with 204 providing mobility data. Participants recorded 27.6 million location points with a median number of 74,865 (IQR 28,471‐186,578) per participant. The mean weekly location points recorded was 95.3 out of 336 possible half-hour intervals (28.4%). Phase II saw more stable data collection in the latter half of the study, due to increased user engagement with the app. Challenges included phone-related issues (screen malfunctions, lost and broken phone), app terminations, and limited internet connectivity. Reverse billing and gamification strategies improved location data collection through increased user engagement.

**Conclusions:**

This study demonstrates that the use of smartphone-based GPS technology is feasible among young adults in a rural South African setting. Although only 28.4% (95.3/336) of expected weekly location data were collected, the study offers insights into engagement strategies that can be used to enhance location data collection in similar contexts. Continuous troubleshooting identified challenges and informed solutions to data collection gaps. Reverse billing system and gamification resulted in significant increases in location data received. These findings underscore the potential of integrating mobile health tools into health systems to better support high-risk mobile populations.

## Introduction

### Mobility and HIV Prevention and Care Outcomes

The scale-up of antiretroviral therapy (ART) has significantly increased adult life expectancy for people living with HIV and reduced HIV transmission [1-3]. Despite this, in 2022, there were 1.3 million new HIV infections and 630,000 HIV-related deaths globally, with the majority occurring in sub-Saharan Africa [4]. Migration, including short-distance and frequent geographic relocations, is sustaining the HIV epidemic [5,6]. Previous studies indicate that highly mobile individuals face a greater risk of acquiring HIV [7-9] and once living with HIV, are less likely to adhere to treatment or achieve viral suppression compared to less mobile individuals [10,11]. Consequently, highly mobile people are at an increased risk of developing AIDS [12] and dying from HIV-related causes [13]. In addition, mobility is linked to high-risk behaviors (eg, multiple and concurrent partners and substance use), which can increase the risk of HIV transmission if individuals are not virally suppressed [14-17]. For example, the treatment and prevention trial in rural KwaZulu-Natal showed that the new HIV infections among transient individuals within the trial communities slowed down efforts to increase ART coverage and population viral load suppression [18]. Therefore, understanding mobility patterns, including destinations, duration, and frequency, is important to inform the development of effective interventions to improve HIV prevention and treatment outcomes.

### Potential of Location-Aware Technologies

Location-aware technologies, such as smartphones, offer simple and accurate tools for capturing both spatial and temporal dimensions of human mobility. A growing body of research has used portable GPS devices, smartwatches, and phones to examine relationships between mobility and health-related outcomes [19-21]. These studies reported sufficient acceptability among participants for using such technologies. However, there were challenges with standalone GPS devices, including satellite signal loss [22], the burden of carrying devices [21], complaints about discomfort or appearance [19], privacy and confidentiality concerns [20], and the need for active user effort to facilitate data collection [23]. Familiar technologies such as smartphones and wearables can mitigate some of these challenges. For instance, potential participants in rural South Africa perceived smartphone GPS valuable and appealing for studying human mobility and HIV risk due to their familiarity with smartphones [24]. Another United States study tested the use of smartphones and smartwatches in chronically ill patients with noncommunicable diseases [23]. Participants preferred smartphones because of ease of use. Also, there was more complete location data from the smartphones compared to smartwatches [23]. However, this study only assessed short-term use (28 days) and focused on noncommunicable diseases, which are typically less stigmatized than infectious diseases like HIV.

We conducted a pilot study to assess the feasibility of using smartphone GPS technology to study linkages between human mobility and poor HIV outcomes among young adults in rural KwaZulu-Natal, South Africa. Young adults in this setting are highly mobile [7] and experience complex and intersecting vulnerabilities (poor risk perception, economic hardships, and lack of social support), increasing their risk of poor HIV care outcomes [14,25]. This paper evaluates the long-term use of smartphone GPS for collecting mobility data among these young adults. We also present key lessons learned from this study.

## Methods

### Study Setting

This study was conducted in uMkhanyakude district, northern KwaZulu-Natal, South Africa, using the Africa Health Research Institute (AHRI) population-based HIV surveillance program. The study area is predominantly rural, with limited access to water and electricity [26]. Internet connectivity is low, mainly accessed via mobile phones on a prepaid basis. Over 85% of youth aged 20‐24 are unemployed [27], and 20% of young adults migrate annually for economic and educational opportunities [7]. The area has high HIV burdens with an incidence rate of 8% among women aged 20‐24 and 4% among men aged 25‐29 years [28].

### Study Design and Dates

**Figure 1.**
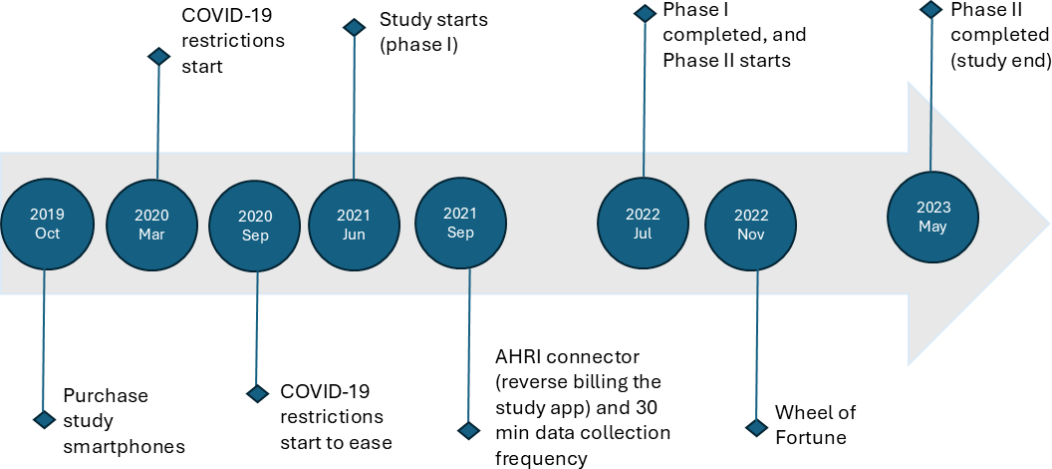
Sesikhona study timelines. AHRI: Africa Health Research Institute.

The study was conducted in 2 phases from June 2021 to May 2023 (see Figure 1). In phase I, participants received an Android smartphone (Proline Falcon XL, Proline, South Africa) with a customized Avicenna app. These smartphones, purchased in October 2019, were chosen for their low cost and compatibility with a proposed app developed for the study. However, this app was found unusable in March 2020, leading to delays in the study to search for an alternative. The COVID-19 pandemic led to further delays, and the study began in June 2021. In phase II, participants used their personal smartphones to address operational challenges faced in phase I.

### Recruitment, Enrollment, and Follow-Up

Individuals were eligible if they: (1) were aged 20 to 30 years old; (2) participated in the 2019 annual AHRI HIV surveillance round; (3) resided in the southern AHRI HIV surveillance area; (4) were willing to participate in the study; and (5) owned a compatible smartphone (minimum random access memory [RAM]: 1GB, sufficient free space for study app installation - phase II only). Eligibility was not limited by HIV status; the study aimed to include both those living with HIV and those who were not. The age criterion excluded individuals aged 18-19 years because they were more likely to be in school, raising parental concerns about smartphone use.

Using the 2019 AHRI HIV surveillance program population as a sampling frame, we generated a random sample of 1467 eligible individuals. Fieldworkers contacted them by telephone to introduce the study, assess eligibility, and interest. Data collection was conducted using Android tablets. Interested individuals were scheduled for a home visit. During the home visit, fieldworkers provided more comprehensive study information, including an informational video addressing privacy and confidentiality concerns identified in preliminary research [24]. The video used animations to explain GPS technology, study procedures, and data protection measures. Participants had the opportunity to ask questions before giving informed consent. After obtaining informed consent, participants were guided through the app installation (phase II) and registration. They were also provided with instructions on the app’s functionality and usage. Participants were followed for 6 months (26 weeks).

**Figure 2.**
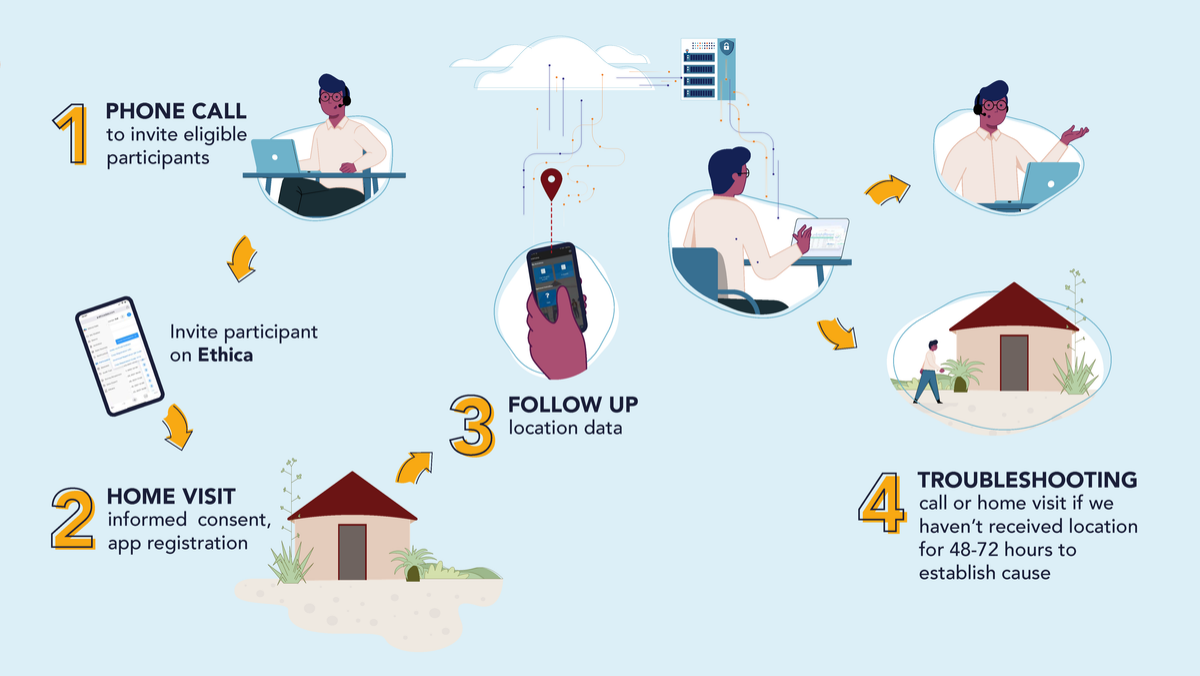
Description of the study enrollment and follow-up process.

Participants received mobile data bundles (phase I: 1GB, phase II: 2GB) at enrollment and monthly thereafter. A portion of the data was intended for app connectivity with the study server, but this was not implemented due to changes with the app service providers. In phase II, monthly mobile was provided only if participants provided location data for at least 10 days. Figure 2 illustrates the study enrollment, participation, and follow-up process.

### Study App and Data Collection

We used the Avicenna software platform [29], customized into a smartphone app called “Sesikhona.” The Sesikhona app, available on Google Play Store, used Android location services to capture GPS coordinates of the device using a combination of Wi-Fi routers, cell towers, and GPS satellites. Initially, the app collected location data every 5 minutes but was later adjusted to 30 minutes (refer to Figure 1), to capture at least 48 location points per smartphone daily. For accuracy, the app recorded GPS data over 60 seconds until 3 precise location points were obtained.

**Figure 3.**
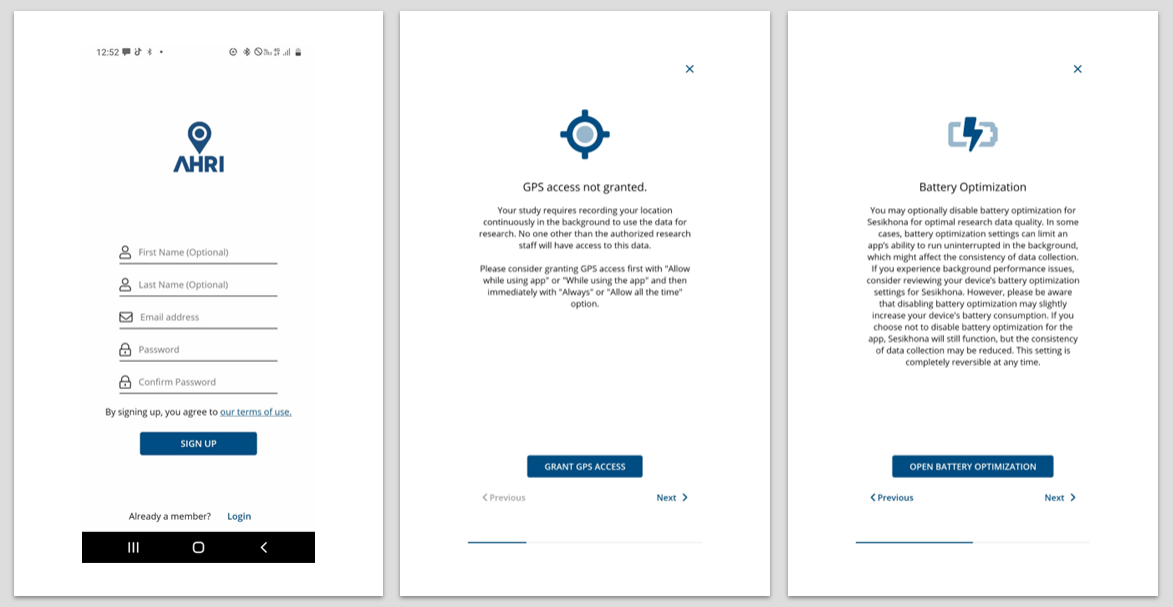
Screenshots of the Sesikhona app showing the registration page, GPS, and battery optimization permissions.

For the app to collect location data, participants needed to: (1) grant the app GPS access; (2) enable location services; and (3) exclude the app from battery optimization (see Figure 3). Participants could revoke these permissions at any time to prevent location data collection. The app uploaded collected location data hourly to a secure study server hosted by Avicenna. If the smartphone lacked internet connection, data was supposed to be stored locally and uploaded during the next scheduled cycle. The data were then transferred from Avicenna to a MySQL database at AHRI.

### App Registration, Troubleshooting, and App Updates

To ensure confidentiality, the app did not collect personal identifying information. Instead, participants were registered with an alias, comprising an AHRI internal number, a study-specific email address, and password. The fieldworker aided the initial app setup by tethering the smartphone to their tablet for internet access. To ensure data integrity, participants were contacted by phone or visited at home if location data was missing for 48‐72 hours. Troubleshooting involved checking app settings such as location permissions, battery optimization, and internet connectivity using REDCap (version 14.044). Fieldworkers used tablets as temporary internet hotspots, enabling the app to resume data transmission.

App updates were managed in 2 processes. Study modifications, such as changes to location data collection frequency, were automatically pushed from the Avicenna platform to participants’ devices in the background. These updates required an internet connection. If offline, participants had to manually initiate the update by selecting the “reload studies from the server” option in the app. Updates to the Sesikhona app, including performance enhancements or bug fixes, were provided through the Google Play Store. Participants had to manually approve and install these updates. Initially, fieldworkers conducted home visits for assistance but later provided remote support via phone calls and data bundles to ensure internet access. App updates were discussed in weekly debriefing meetings with the project operations team, reviewing recruitment, enrollment and follow-up progress, technical challenges, and study procedures. Notes from these meetings informed revisions and improvements.

### Data Analysis

We report on participants’ enrollments, key demographics, HIV status at enrollment, age group, education years, and socioeconomic status. Feasibility was assessed using collected location data, including the number of location data points and weekly averages per participant. Also, we reviewed project operation meeting notes to construct a narrative of our study implementation experiences. This review highlights challenges in data collection, like phone replacement and device settings, and strategies to improve data collection.

### Ethical Considerations

The study was designed with special attention to ethical considerations related to location data collection. We conducted formative research and applied the Emanuel ethical framework to identify the ethical issues and used the findings to inform the development of the informed consent materials and overall study design [24]. The study was approved by the Biomedical Research Ethics Committee of the University of KwaZulu Natal (BREC Ref 460/15). All participants provided written informed consent before participation, and study participation was entirely voluntary. To ensure privacy, all data were deidentified, with no personal identifying information in the final dataset. Participants received smartphones in phase I to keep after the study, along with data bundles in both phases as incentives for participation.

## Results

We first describe participant characteristics, then summarize the collected location data. Finally, we discuss the operational challenges faced and opportunities identified during the study implementation.

### Enrollment and Participant characteristics

A total of 516 individuals were contacted (including 88 in phase II), of whom 207 consented to participate (phase I: 163 and phase II: 44). Of the 207 participants, 3 did not provide location data (Table 1): one due to incomplete registration and 2 due to device incompatibility. Among those who provided location data, 131 out of 204 (64.2%) were female. Most participants, 95.2% (160 out of 168 that reported education) completed 10 to 17 years of education, and 39 out of 204 (19%) were living with HIV. Regarding age distribution, 80 out of 204 (39.2%). However, phase II participants were slightly older with a higher socio-economic status (Table 1).

**Table 1.** Demographic characteristics of study participants^a^.

Characteristic	Phase I (n=162), n (%)	Phase II (n=42), n (%)	Total (n=204), n (%)
Sex			
Female	99 (61.1)	32 (76.2)	131 (64.2)
Male	63 (38.9)	10 (23.8)	73 (35.8)
HIV status			
Negative	129 (80.1)	35 (83.3)	164 (80.8)
Positive	32 (19.9)	7 (16.7)	39 (19.2)
Missing	1	0	1
Age			
20‐24	68 (42.0)	12 (28.6)	80 (39.2)
25‐30	94 (58.0)	30 (71.4)	124 (60.8)
Years of education			
[0,10]	7 (5.4)	1 (2.6)	8 (4.8)
[10,17]	123 (94.6)	37 (97.4)	160 (95.2)
Missing	32	4	36
Socioeconomic Index^b^			
[1–2]	7 (4.5)	3 (7.3)	10 (5.1)
[2–3]	103 (66.9)	21 (51.2)	124 (63.6)
[3–4]	44 (28.6)	17 (41.5)	61 (31.3)
Missing	8	1	9

a Excluding the 3 that did not contribute location data.

b Socioeconomic index with [1-2] representing very low socio-economic status, [2-3] low socioeconomic status and [3-4] lower-middle socioeconomic status.

### Location Data

**Figure 4.**
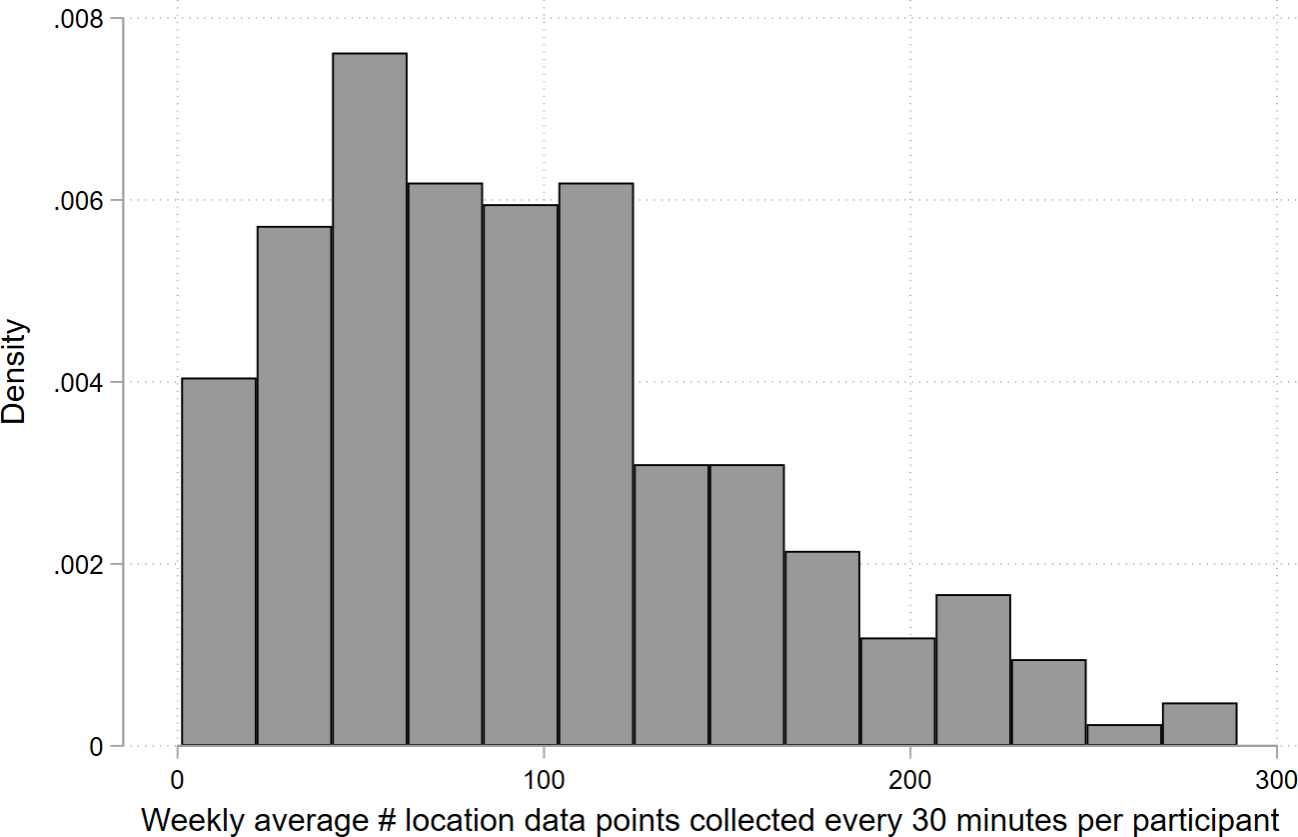
Range and density of weekly location data.

A total of 204 participants recorded 27,589,855 location points, with a median number of 74,865 (IQR 28,471‐186,578) points per participant. Participants provided a median of 2906 (IQR 856‐6688) location points weekly, though consistency varied. Ideally, location data were collected every 30 minutes, equating to a minimum of 336 location points weekly (48 daily). However, on average, participants collected 95.3 out of the expected 336 points (28.4%) weekly. The range and volume of location data varied by participant (Figure 4).

**Figure 5.**
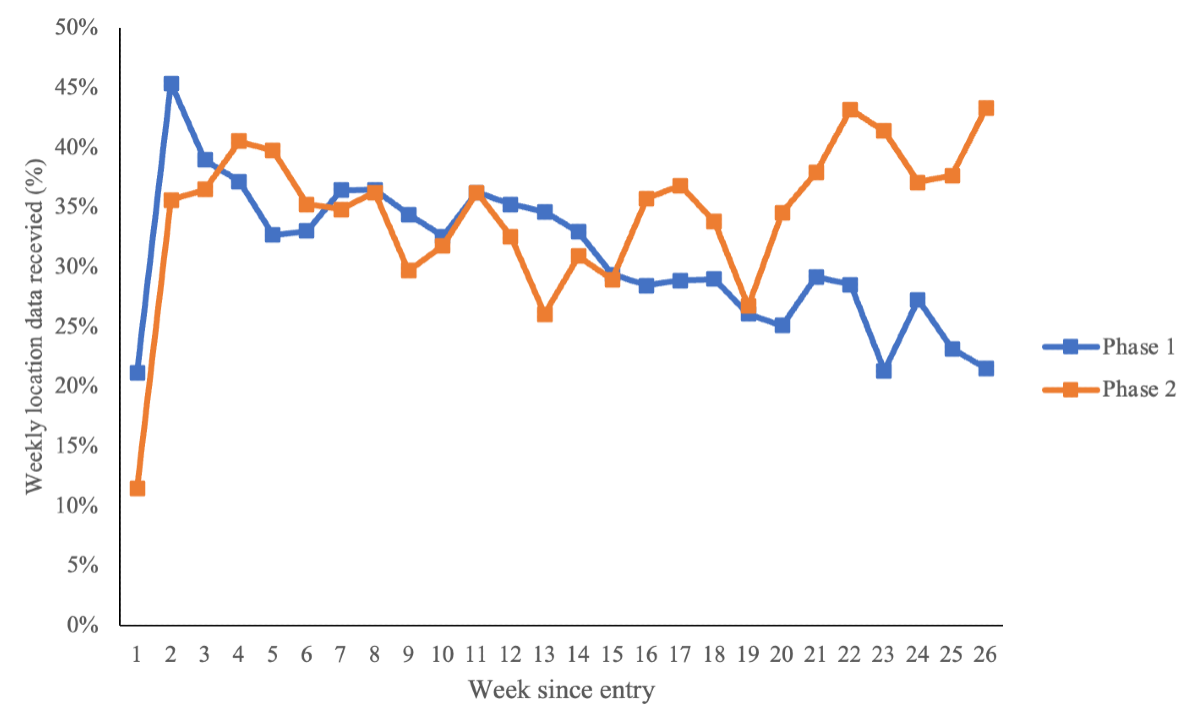
Mean percentage of location data points received per week by phase.

In phase I, weekly location data peaked at 45.4% (SD 29.7%; 152.5/336) in Week 2 and declined by more than half to 21.5% (SD 22.2%; 72.3/336) by Week 26. In phase II, data collection fluctuated with participant engagement, increasing in the latter half of the study and peaking at 43.3% (SD 36.5%; 145.5/336) in Week 26. However, the overall difference between the 2 phases was 28.3% (SD 15.6%; 95.0/336) versus 29.0% (SD 26.6%; 97.4/336) was not statistically significant (*P*=.82) using a 2-sample *t* test. (Figure 5).

### Operational Challenges and Opportunities

Several operational challenges emerged during phase I of the study. A major issue was the app’s reliance on internet connectivity to upload location. In the first month, we found that many participants quickly exhausted their prepaid data bundles, leading to gaps in location data. To address this, we partnered with Datafree technologies [30] to implement a reverse-billed app (AHRI connector) which allowed the Sesikhona app to upload collected location data without an active data bundle on participants’ devices. However, deploying the AHRI connector app (available as an APK file) required home visits to install via Bluetooth. While this was a logistical challenge, it presented an opportunity to initiate the study update to the increased 30-minute data collection frequency. Reverse billing initial data bundle balance requirements were bypassed by tethering participant devices to the fieldworker tablet. Another challenge stemmed from hardware limitations. The study smartphones, purchased in 2019, faced significant technological issues due to their prolonged storage and aging hardware. Common malfunctions included frozen touchscreens, loss of network signal, and hardware failures, resulting in 77 phone replacements, with some 20 participants requiring multiple replacements (refer to Multimedia Appendix 1). Some participants lost or broke their phones. Notably, 16 participants opted to install the app on their personal phones, demonstrating early acceptability of personal phone use for the study.

In addition, the app was prone to frequent app terminations resulting in missing location data. Despite participants maintaining correct app settings, including 90% (324/360) with location services enabled (on), 95.5% (345/361) with battery optimization disabled, and 76.8% (274/357) with AHRI connector on (refer to Multimedia Appendix 2), the app was not collecting location data. Subsequent analysis by the Avicenna team determined that these disruptions were due to the smartphone operating system background task management, which automatically terminated apps running in the background, including Sesikhona, to optimize system performance for active apps.

### Adaptations and Phase II

In response to the phone-related challenges and participants’ preference for switching to personal phones, phase II adopted a revised approach. Unlike phase I, where the study provided smartphones, phase II required participants to install the Sesikhona app on their own devices. Of the 44 participants who consented, 2 had smartphones with 512 MB RAM, insufficient for the app. This issue was not initially identified, but discussions with Avicenna established that at least 1 GB RAM was needed for optimal app performance. Similarly to phase I, participants reported losing (n=2) or breaking (n=2) their phones, and 8 participants switched to different phones. In addition, a few participants changed their phone numbers. Nine participants deleted the app across both phases (5 in phase I and 4 in phase II), 6 reinstalled it, and 3 were no longer interested. Reasons for deletions included unintentional removal by children, app performance issues, or insufficient storage. Some intended to reinstall but were unable to due to forgotten login details. Fieldworkers provided reinstallation assistance and technical support to minimize data collection disruptions.

**Figure 6.**
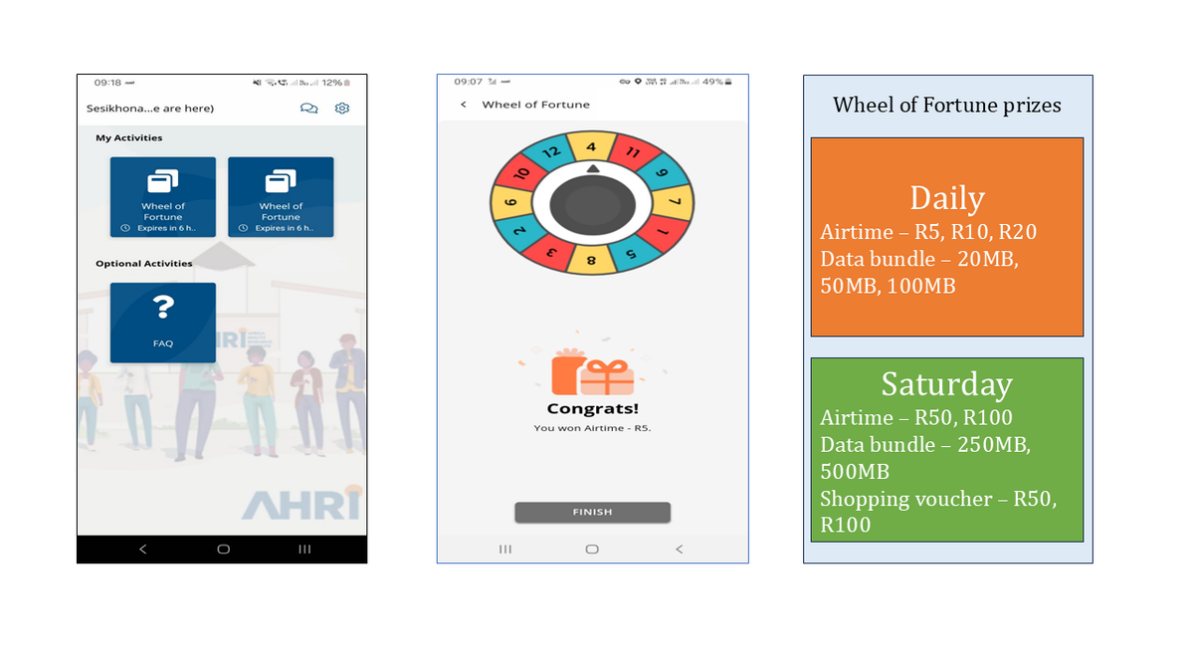
Wheel of Fortune aimed to increase user engagement with the app. Screen 1: how it appears on the Sesikhona app homepage; screen 2: spinning wheel with an example of a win; and screen 3: the prizes.

Location data gaps persisted in phase II despite correct app settings. To enhance user engagement and reduce app terminations, we introduced a gamification (Wheel of Fortune) in November 2022. This daily lottery game (available from 9 AM to 4 PM) with additional plays on Saturdays offered participants an opportunity to win prizes like airtime, data bundles, and shopping vouchers (see Figure 6).

Engaging with the game was positively correlated with the frequency of location data collection. The probability of participants providing location data was 77.6% days when they played the game, versus 27.5% if they did not play (refer to Multimedia Appendix 3). The odds ratio was 9.12 (95% CI 5.44‐15.29) with a *P*<.001. In addition, playing the game was associated with +15.7% (7.5/48*; P*=.007) increase in the amount of location data received per day (refer to Multimedia Appendix 4).

Table 2 summarizes the key findings from phase I and II. While phase II had few participants, having AHRI connector at the outset and introduction of the gamification enhanced user engagement and data collection. Overall, there were fewer technical challenges reported in phase II compared to phase I.

**Table 2.** Summary of phase I and phase II results.

Parameter	Phase I (June 2021 to July 2022)	Phase II (July 2022 to May 2023)
Participants enrolled, n	163	44
Participants contributing location data, n	162	42
Average number of location points/participant, median (IQR)	67,398 ( 29,771‐168961)	47,510 (12,252‐172,613)
Range of location points/participant	389‐670,384	53‐950,597
Percentage of location points per week (out of 336 half-hour intervals), mean (SD)	28.3 (15.6)	29.0 (26.6)
Introduction of AHRI^a^ connector (reverse billing app)	Implemented midway	Implemented at the start of the phase
Phone replacements	77 phones replaced due to technical issues	2 participants’ phones were incompatible, with 4 reporting broken/lost phones
Gamification (Wheel of Fortune) impact	N/A^b^	Increased probability of receiving location data by 77.6% if the participant played the game, versus 27.5% if they did not play and associated with +15.7% more data received in a per day compared to not playing (*P*=.007)
Challenges faced	Aging hardware, data bundle exhaustion, app termination	Some phone compatibility issues, but fewer technical problems overall
Engagement strategy	Relied on troubleshooting and participant support	Gamification introduced to enhance engagement and data collection

a AHRI: Africa Health Research Institute.

b not applicable.

## Discussion

### Principal Findings

In this study, we found that smartphone-based GPS technology is a feasible approach for studying human mobility in rural settings using commercial software, provided that key implementation challenges are addressed. Individuals living with HIV and without accepted both study-provided and personal smartphone use for location data collection. Although most participants complied with study protocols, only 28.4% (95.3/336) of the expected weekly location data were collected, largely due to operational challenges such as hardware malfunctions, lack of internet connection, and frequent app terminations, particularly during phase I. Frequent troubleshooting provided insights into context-specific adaptations needed for rural settings, including implementation of the reverse billing to facilitate data transmission with an active data bundle.

The introduction of gamification in phase II significantly increased the likelihood of receiving location data from participants who engaged with the game. This highlights the potential of gamified strategies to enhance participant engagement and improve data completeness in long-term studies. Notably, phase II participants were slightly older and from higher income brackets, raising important considerations for equity in future studies. Requiring smartphone ownership could exclude younger or lower-income populations, who may be at higher risk for HIV and stand to benefit most from mobility-informed interventions.

### Comparison to Prior Work

Our results align with previous studies demonstrating feasibility and acceptance of smartphone GPS apps to understand HIV risk [31-33], substance use and violence among people living with HIV [34]. The lower median data collection of 28.4% highlights challenges in rural settings. Two South African smartphone-based studies reported significant technological and operational issues with using personal phones, where inadequate RAM, storage, and device type affected implementation [35,36]. Furthermore, Clouse et al noted how lack of internet contributed to missing location data [36].

Although Hardy et al [23] found that smartphones provided more complete location data with less user effort, our study showed that the app required significant user effort to collect data. The high number of days with missing location data, especially later in the study, is comparable to findings from studies using standalone GPS devices [20,21]. However, these studies had smaller sample sizes, shorter follow-up periods, and different settings. Finally, other research shows that game elements enhance user engagement in mobile apps [37].

### Implications for Future HIV Research and Interventions

The mobility data from this study will provide a granular understanding of how mobility intersects with HIV risk. Identifying geographic hotspots and mobility patterns linked to high-risk behaviors, such as multiple concurrent partnerships or inconsistent condom use, can enable more targeted and efficient allocation of prevention resources. For example, interventions can be tailored to specific locations and times where risk is highest to maximize impact.

Mobility data can also reveal disruptions in HIV care, such as missed clinic appointments due to travel, informing the design of tailored interventions like mobile clinics or targeted outreach programs at critical locations. Integrating mobile health tools, like the Sesikhona app, into broader health systems offers opportunities to prospectively monitor human mobility and deliver real-time support to individuals at risk of disengagement. For example, automated reminders or check-ins could be triggered on an individual’s location to encourage care engagement or treatment adherence protocols. However, for these strategies to be effective, technological and access barriers must be addressed, and the interventions should be co-designed with communities to align with privacy expectations and ethical standards. Overcoming these challenges will be critical to leveraging mobility-informed approaches to achieving the UNAIDS 95-95-95 targets, particularly among highly mobile and hard-to-reach populations.

### Study Strengths and Limitations

This study provides important evidence on the feasibility of using commercially available smartphone GPS apps to collect mobility data in a rural, resource-limited setting. While the high adoption rate and engagement demonstrate potential, feasibility was constrained by technological and operational challenges related to device and internet connectivity. These findings suggest that use of smartphone-based GPS to study mobility is feasible if key barriers are addressed. However, generalizability may be limited. The study was conducted within a cohort already engaged through the AHRI HIV population-based surveillance program, where established trust likely enhanced participant acceptance. In addition, reliance on smartphone technology may have introduced access and literacy barriers, which could affect scalability in other settings.

### Conclusion

This study provides critical insights into the feasibility of using smartphone-based GPS technology to collect mobility data among young adults in a rural, HIV-hyperendemic setting in South Africa. By comparing the outcomes of 2 distinct phases, the study highlights the success and challenges of using both study-provided and personal smartphones. Although the collected data was lower than expected, high adoption rates, sustained engagement, and the success of solutions such as reverse billing and gamification demonstrate the potential of smartphone-based approaches to study mobility, provided technological barriers are addressed.

The implications of this study extend beyond its immediate context, offering valuable lessons for the broader field of HIV research and intervention. Our findings underscore the potential of integrating mobile health tools into health systems to support high-risk mobile populations. Mobility-informed approaches could enhance real-time monitoring, targeted service delivery, and ultimately improve HIV prevention and care outcomes. Further research is needed to refine engagement strategies, address technological and access barriers, and optimize data integration methods to fully leverage mobility data for achieving global HIV epidemic control.

## Supplementary material

10.2196/67519 Multimedia Appendix 1 Phase I phone replacement reasons.

10.2196/67519 Multimedia Appendix 2 Phone settings during troubleshooting.

10.2196/67519 Multimedia Appendix 3 Probability of receiving location data on a given day.

10.2196/67519 Multimedia Appendix 4 Expected percentage of data received (out of 48 daily half-hour blocks).

## Abbreviations

AHRI: Africa Health Research Institute
ART: antiretroviral therapy
RAM: random access memory
