# Supplementary figures and images for: Leveraging Smartphone Mobility Data to Understand HIV Risk Among Rural South African Young Adults: Feasibility Study
Source: JMIR Mhealth Uhealth. 2025 Aug 25;13:e67519. doi: 10.2196/67519 (PMC12377519; doi:10.2196/67519)

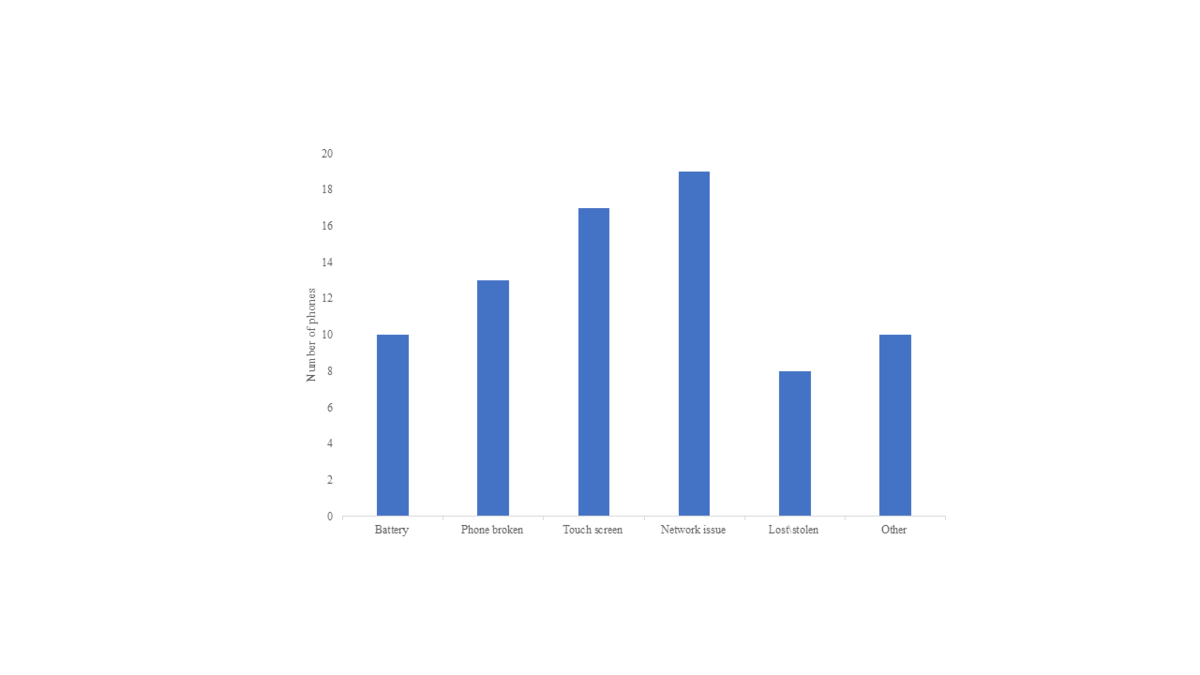

Supplement: Multimedia Appendix 1 [file mhealth-v13-e67519-s001.png]

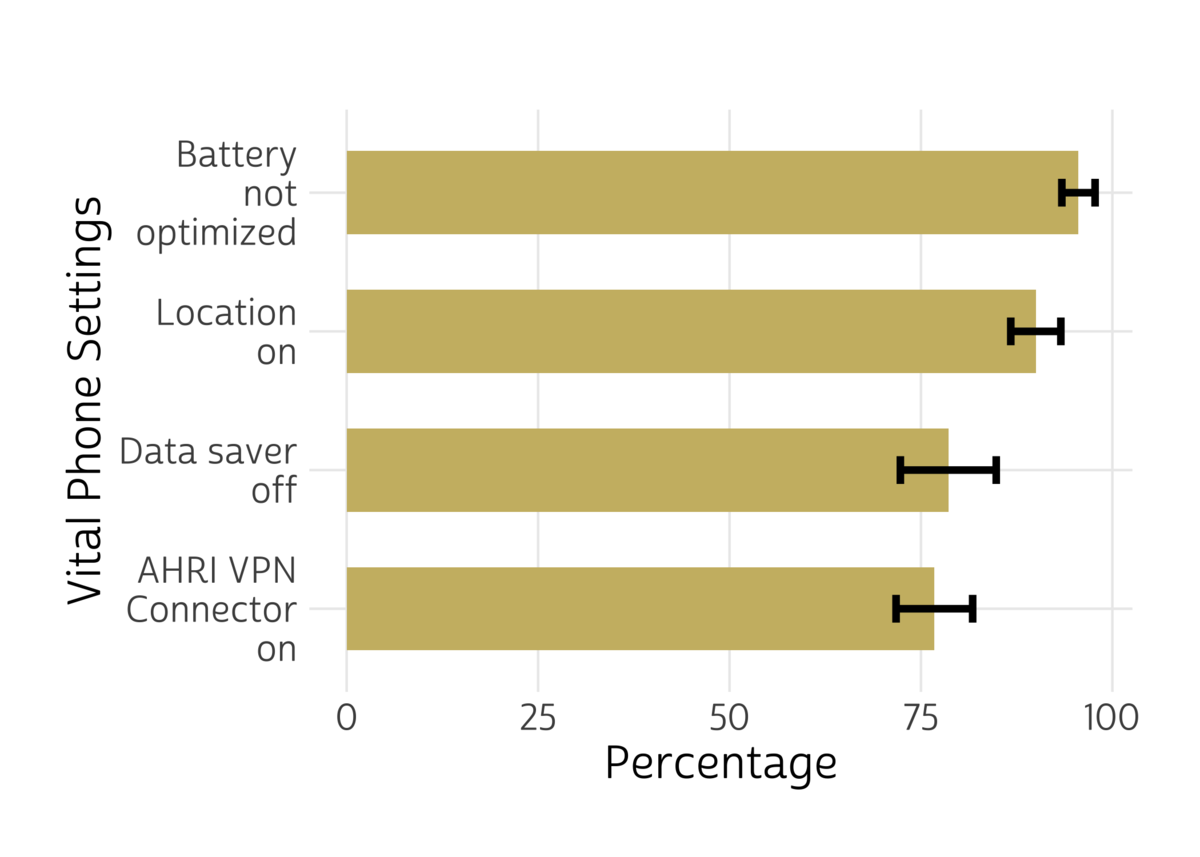

Supplement: Multimedia Appendix 2 [file mhealth-v13-e67519-s002.png]

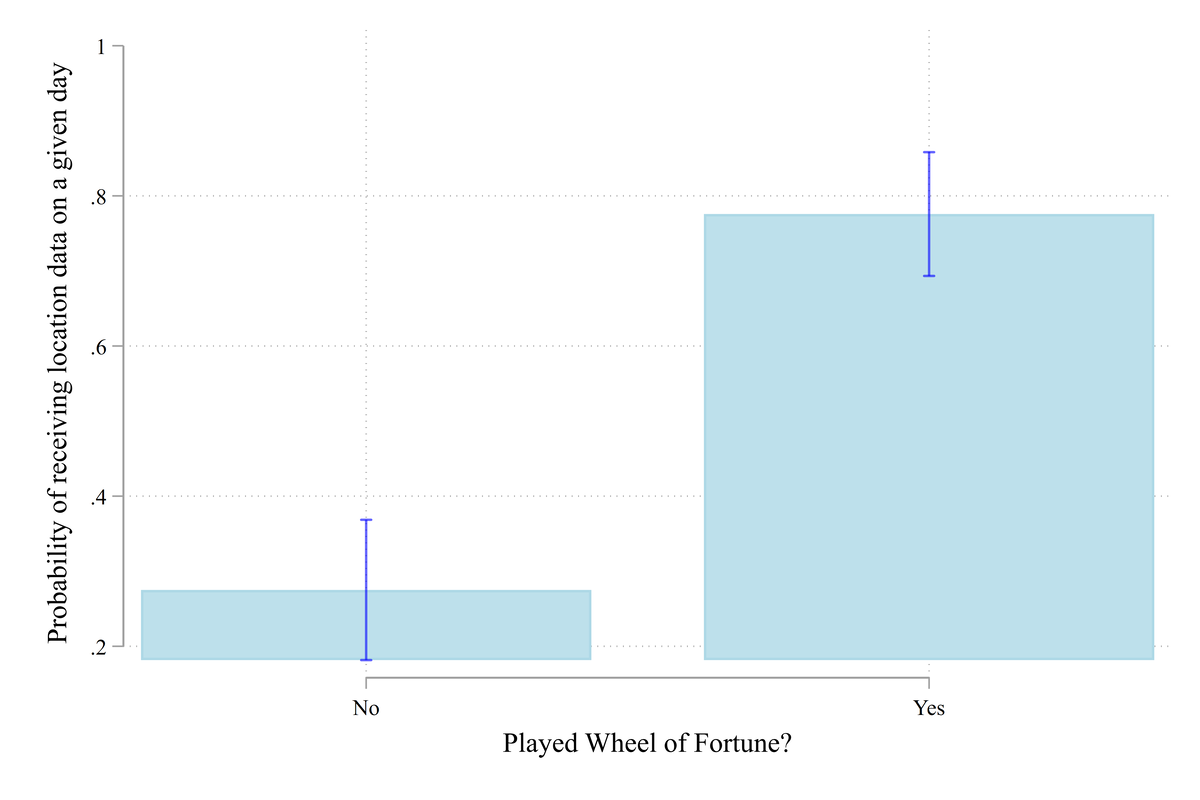

Supplement: Multimedia Appendix 3 [file mhealth-v13-e67519-s003.png]

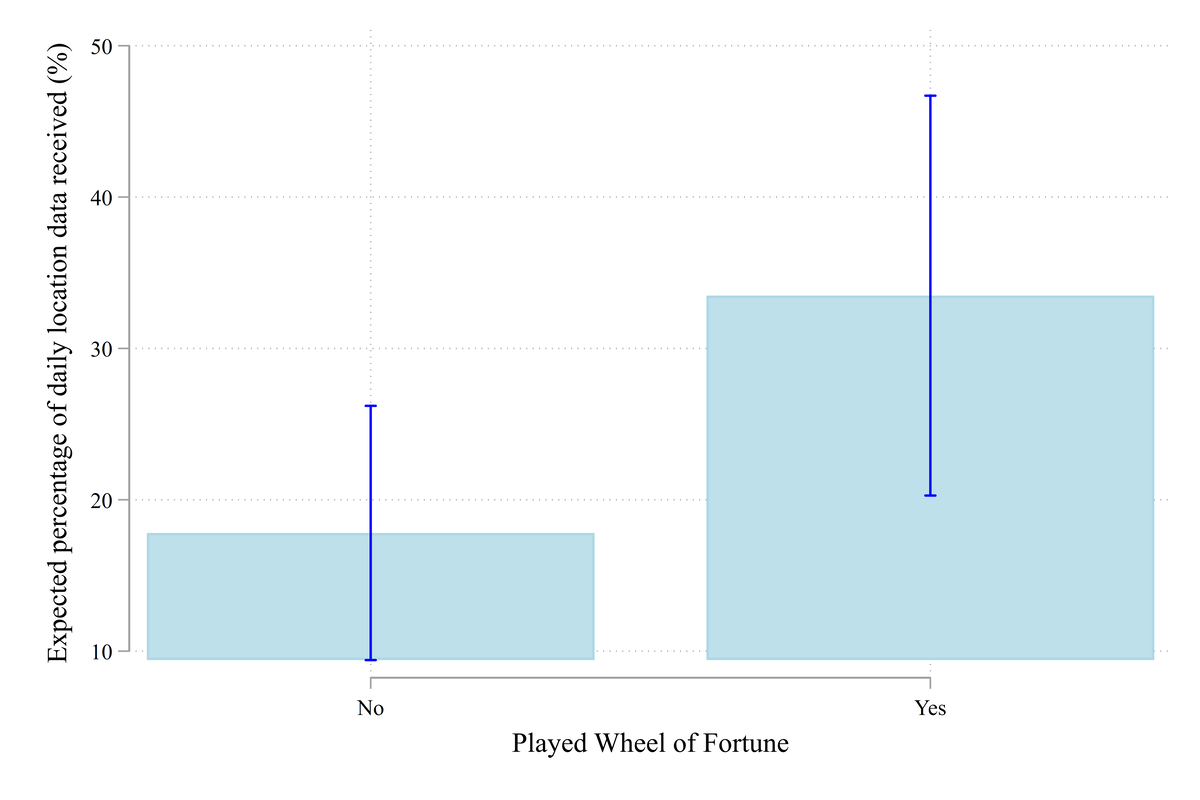

Supplement: Multimedia Appendix 4 [file mhealth-v13-e67519-s004.png]
